# Central Dental Association of Northern New Jersey

**Published:** 1893-03

**Authors:** 


### CENTRAL DENTAL ASSOCIATION OF NORTHERN NEW JERSEY.

### DISCUSSION ON DR. SWIFT'S PAPER.

(For this paper, see page 168.)

*Dr. Hardy.*—Dr. Swift's method I think is a very good one, and proves very successful; at least I find it so. In treating cases of this kind it is better to leave the application there and give the tooth sufficient time to get well before disturbing it again. I do not think you should remove the application in two or three days, for this keeps the tooth in a constant state of irritation; a healthy tooth constantly disturbed becomes more or less irritated. Dr. Swift speaks of treating abscesses by forcing the liquid medicament through the tooth. Instead of that method, I believe in filling the canal entirely and treating the abscess from the outside with peroxide of hydrogen.

*Dr. Fish.*—This is a subject which possibly no two men in this room to-night can agree upon. In treating these cases I think we have to decide on absolute cleanliness. No matter what course we pursue, whether by coagulants or by the use of essential oils, we arrive at that one point, and in that we will have success.

The essayist claims that in his experience he finds that the essential oils, owing to their rapid diffusibility, will penetrate deeper into the tubuli, and retain, as I understand it, their disinfectant properties longer than an escharotic which is forced into the tooth, and which he claims dams up the tubuli. I cannot say that I agree with him, for the reason that the same identical results have been attained by the use of carbolic acid as that said to be secured by eucalyptus, oil of cassia, and the various other agents mentioned in this paper.

If I wished to place myself in the position of a plagiarist, I might go on and give you a long discourse about my treatment of putrescent pulps, but I have only been in practice three years, and during that time have had very little opportunity to judge of the lasting results of any method which I have pursued. I can only repeat what I said before, that in my opinion the object to be aimed at is absolute cleanliness, be it reached with hot water, bichloride, peroxide of hydrogen, or any other remedy; the fundamental principle rests there.

The essayist, I think, makes an error when, in the treatment of these teeth, he advises the leaving a vent to the cavity. I do not care what antiseptics are placed in a tooth, if he leaves the cavity exposed by a small canal he has a place for septic agents to enter and set up future trouble.

I have found that coagulants work well; I have had better success with the use of these than I have had with the essential oils. And in connection with the treatment, my method is to first clear the roots out thoroughly, and syringe with a solution of bichloride,—about 1 to 500,—using due care not to force anything through the foramen; broaches are not used, or anything that will act as a piston, which is a most powerful instrument to force agents through the end of the root. I should prefer to use a hypodermic needle, previously removing all the septic matter from the root and drying it thoroughly and wiping it out with coagulants, then using a solution of ether and iodoform. I must admit that there are a great many who object to this method. Possibly some of them may have grounds for the antagonism, but I very much doubt whether there is any solid basis for this. I use a solution. It is very diffusible, and has all the essential elements of a good disinfectant. After treating the canal, I dry it out and fill with chloro-percha, or other filling of that material. So far as I have been able to observe I have had a very good percentage of success.

*Dr. George Evans.*—Mr. President, I had no idea of talking to-night, but there are one or two points that I may refer to. The subject is, I believe, putrescent pulps, and I will endeavor to discuss the paper, rather than the remarks of other gentlemen who have spoken upon it.

The essayist enforces the necessity of preventing the saliva from entering the pulp-canal. That is a point which is often brought forward. That is very sensible advice in the case of a root-canal from which you have taken a freshly-destroyed pulp, but it makes very little matter whether or not the saliva is permitted to enter a cavity while there is a putrescent pulp present or just removed. What is worse than a putrescent pulp? Is saliva? Not at all. After you have cleared out the canal and commenced your antiseptic treatment, keep out the saliva; but at the beginning of the operation it is almost needless to discuss the question. I believe thoroughly in the use of essential oils. Carbolic acid and creosote have their place in dentistry; they are old-fashioned remedies. Now, it is an undisputed fact that carbolic acid has not the diffusible power in a pulpless tooth possessed by the essential oils, especially oil of cassia. I believe that Dr. Miller in his experience has demonstrated the fact that the oil of cassia, or cinnamon oil, is a more powerful antiseptic agent by far than is carbolic acid.

Iodoform is considered at the present time in dental practice, by those who are studying new methods of treatment and new agents, as one which is ready to be laid aside on the shelf in dental practice; first, because iodoform has no greater power as an antiseptic agent than aristol, and because it is a most disagreeable one to use in the dental office. You can take a saturated solution of any one of the essential oils with aristol and get all the effect of iodine. Place cotton in the canal with a saturated solution of aristol; it is agreeable to the patient, and its action as an antiseptic agent is far superior to that of carbolic acid or creosote. Carbolic acid and creosote differ materially as agents for use in pulpless teeth. Carbolic acid is soluble in water, while creosote is not so readily miscible; and if you fill a pulpless tooth with a preparation of carbolic acid, and seal that up as closely as you can and in any form you wish, or pack it solid if you please, and dismiss the patient for a year, and then open that root-canal and take out that cotton, there will be but little odor of carbolic acid, it having been absorbed in some way, or passed from the root-canal; but where the essential oils are used, mixing them with some one of the various preparations of iodine, there is a permanent antiseptic agent in the root canal, which establishes and maintains an aseptic condition.

The essayist spoke of using a trephine. I am decidedly opposed to that treatment, because if the operator can always tell where the abscess lies, and just where the apex of the root is, he is very expert, more expert than I am, and I think more so than the majority of practitioners. And, aside from the uncertainty of reaching the point aimed at, the operation is a severe one. There is one thing that I cannot understand,—that is, how it is that so few practise the admirable method of entering into the apical space, presented by Professor Black; that is, the use of carbolic acid along with the instrument, and exfoliating the tissue. In that way I can enter the apical space with a minimum amount of pain to the patient. It is performed in this way: The gum is dried, and then with a plugger a drop of carbolic acid is placed directly over the place where the apical space is supposed to be, letting it rest a moment; then commence removing with an instrument the tissue. In a few moments apply another drop of acid and remove as before, repeating this from time to time, and in a short time the alveolar process can be penetrated, and with a burr drill make the entrance direct to the apex of the root.

*Dr. Watkins.*—Mr. President, I would like to ask Dr. Evans when Dr. Black published that method of using carbolic acid in opening an abscess; about how long since. I thought I was the originator of that method. I described that method before the First District Dental Society of New York eight or nine years ago, and it was published in the *Dental Cosmos.* Up to that time I had never heard it mentioned or seen it in print.

*Dr. Evans.*—So far as I can I like to give honor to whom honor is due; no one wants to give it where it is merited more than I do. I was not aware that Dr. Watkins had introduced that method. I referred it to Dr. Black, in a measure, because he has presented it in the "American System of Dentistry," and he has not given Dr. Watkins credit for it.

*Dr. Luckey.*—I think the credit of introducing this method belongs to our old friend Dr. Atkinson, who performed the operation in New York at least fifteen years ago, and described it many times since.

*Dr. Evans.*—I have only one word more to say, and that is, if this method is so well known, why is it not practised more?

*Dr. Swift.*—I have used carbolic acid in the manner described for the past seven years, and also attributed its origin, as Dr. Luckey does, to Dr. Atkinson.

*Dr. Ottolengui.*—I hope you will pardon my saying that this contention over the name of the originator of any method is a waste of time. It is the method that we want, and not the name of the originator. It does not make much difference to the patient who discovered a particular method so long as the man who is operating knows how to use it. With regard to this process, it seems to me that you are claiming something for very little; because, after all, you only penetrate to the alveolar process with it; passing through the gum-tissue, which is not very sensitive, and then enter the bone, which is not so responsive to the carbolic acid; and after that enter the apical space, the point where pain will be felt; it is the inflamed tissue at the end of the root, the pericementum, that will make the response, and unless the carbolic acid acts upon that and removes the sensitiveness, time is wasted in using it on the gum.

I find that I cannot get along without an ethereal solution of iodoform. And I desire to say that the ethereal solution of iodoform has not the bad odor generally represented. And another curious thing about iodoform is this: I have seen it stated, I think by Dr. Miller, that iodoform is not a germicide to any great extent; nevertheless if you go into a hospital ward you will see five or six yards of iodoform gauze and no carbolic acid. I think if it is good enough for major surgery it is good enough for the minor operations in dentistry. It is not wise to run after every new drug that is put on the market, thinking it is better than anything else that was ever introduced. As long as iodoform serves its purpose it is a good thing.

I want to say a word about the success that has been reported as obtained from the various methods of filling or treating teeth. It is wonderful what an amount of bad work you can do to the teeth and have them stay there. Whether coagulants or non-coagulants be used, a large majority—I think over eighty-five per cent.—of the teeth will stay in any way; if you do the work with passing skill it will remain, and the teeth will be retained in the mouth until they become abscessed; and who is to know how long it will be until this occurs? There are two classes of pulpless teeth, one in which eighty-five or ninety per cent. of successes are obtained; the roots of these can be entered and without much care. Then there is a separate class having those minute canals in the buccal roots and the anterior roots of lower molars, and I think they make up the other fifteen per cent. I suggested lately, at a meeting in New York, that it might be possible that after the pulps had been taken from these buccal roots we might find some way of leaving the rest in; of course we can leave them, but let us see if we can leave them there with safety. I suggested that perhaps treating liberally with choloride of zinc might mummify or coagulate them. I tried this on a case recently. I removed the usual percentage of the pulp from the palatal root-canal, and did not get anything out of the other two canals. I put in chloride of zinc and left it in for several days, and to my surprise I was able to go a little way up in these buccal roots; how far proportionately I don't know, but I did go so far that I could see the three openings in the canals. I treated once more with chloride of zinc, and at the next sitting filled these canals as far as I could, and in five days afterwards there was a fistulous opening over the buccal canal. The suggestion in that case did not amount to anything.

*Dr. Watkins.*—I would like to say, before this subject is passed, a word in favor of carbolic acid in the treatment of putrescent pulps. Everything we hear now seems to be in opposition to its use. All have used it, and I keep it in my office, and I use it more than any other medicine, and have more success with it than with any five other compounds. If there is one other agent I want in my office it is iodoform; and in many cases I mix them together and insert them, as in the treatment of putrescent pulps. In a week or ten days after this the tooth is ready to fill without any further treatment. I believe that if the canal be opened and treated thoroughly the first time, in nine cases out of ten that will suffice.

*Dr. Sanger.*—I would like to say a word in reference to Dr. Evans's root-dryer. I have heard a number of discussions on the subject of putrescent pulps without hearing this instrument spoken of. In my own practice it is invaluable in cases where access to the pulp-chamber is secured. My practice is to follow what is called the immediate root-filling process, and to do that I have always had recourse to this. Having removed all of the pulp that is possible, I proceed to dry out the root until I get no odor at all It requires a little patience sometimes, but after thoroughly using this dryer I can close a canal and retire with a clear conscience.

*Dr. Eaton.*—I am of the opinion that if Dr. Watkins would try eucalyptus instead of carbolic acid, in combination with iodoform, he would find that he would get equally as good success if not better. I have been in practice for nearly thirty years, and I don't know how it is, but somehow I have always managed to get along without opening into the apical space. Perhaps I do not do as well as others, but I always get along without doing that. If I have a fistula I treat it through that channel.

On motion, the subject was passed, with the thanks of the Association to the essayist.

